# Trichinella pseudospiralis outbreak in France.

**DOI:** 10.3201/eid0605.000517

**Published:** 2000

**Authors:** S Ranque, B Faugère, E Pozio, G La Rosa, A Tamburrini, J F Pellissier, P Brouqui

**Affiliations:** Hopital F. Houphouët Boigny, Marseille, France.

## Abstract

Four persons became ill with trichinellosis after eating meat from a wild boar hunted in Camargue, France. Nonencapsulated larvae of Trichinella pseudospiralis were detected in meat and muscle biopsy specimens. The diagnoses were confirmed by molecular typing. Surveillance for the emerging T. pseudospiralis should be expanded.


### *Trichinella pseudospiralis* Outbreak in France

**Stéphane Ranque,* Bernard Faugère,*† Edoardo Pozio,† Giuseppe La Rosa,† Alessandra Tamburrini,† Jean-François Pellissier,‡ and Philippe Brouqui***

*Hopital F. Houphouët Boigny, Marseille, France; †Instituto Superiore di Sanità, Rome, Italy; ‡Laboratory of Neurological and Muscular Biopathology, Marseille, France

Four persons became ill with trichinellosis after eating meat from a wild boar hunted in Camargue, France. Nonencapsulated larvae of *Trichinella pseudospiralis* were detected in meat and muscle biopsy specimens. The diagnoses were confirmed by molecular typing. Surveillance for the emerging *T. pseudospiralis* should be expanded.

Until 1995, *Trichinella pseudospiralis*, a nonencapsulating species of the genus *Trichinella* and the only species that infects both mammals and birds, was not considered a potential pathogen of humans and domestic animals, since it had been detected only in sylvatic animals (raccoon dog, corsac fox, tiger cat, tawny eagle, and rook) in remote regions (Caucasus, Kazakhstan, Tasmania) (1). However, this pathogen has since been detected in wildlife in the United States (2), in domestic and synanthropic animals and humans in Russia (3), and in humans in Thailand (4). In October 1999, a human outbreak of trichinellosis in France was traced to infected wild boar meat. We describe the epidemiologic, clinical, and laboratory investigations that confirmed *T. pseudospiralis* as the etiologic agent.

### The Study

In October 1999, four adults living in Miramas, a small town in southeastern France, sought medical attention for asthenia, fever, nausea, and watery diarrhea. Three of the patients were members of the same household (father, mother, and son); the fourth was a friend. Their family physicians prescribed symptomatic therapy for gastroenteritis, but their conditions worsened and they were referred to the Infectious and Tropical Diseases Unit of a teaching hospital in Marseille. In initial interviews, all four patients said that they had eaten undercooked barbecued wild boar *(Sus scrofa)* meat on October 7. The father and his friend hunted the boar in Camargue, a swampy region in the Rhone River Delta.

Frozen wild boar meat was thawed, artificially digested, and washed (5) to obtain *Trichinella* larvae from muscle tissue for examination. Individual larvae were suspended in 5 µl water and stored at -30°C. Larvae were identified by polymerase chain reaction (PCR) analysis in which 10 µl 0.1 M Tris-HCL, pH 7.6, was added to the larvae, overlaid with mineral oil, and heated at 90°C for 10 minutes. PCR was done with *Taq* DNA polymerase, 10X PCR buffer, and deoxynucleoside triphosphates (dNTPs) (Takara, Otsu, Shiga, Japan). The 30-µl PCR cocktail contained 10X PCR buffer at a final concentration of 1.5 mM $MgCL_2$, 200 mM dNTPs, 50 pmoles of each primer, and 0.5 unit *Taq* DNA polymerase. For amplification, 2 µl of a single larva preparation was used. Amplifications consisted of 35 cycles as follows: denaturation at 94°C for 20 seconds, annealing at 58°C for 1 minute, and elongation at 72°C for 1 minute. The primer set oTsr1 (5'-CGA AAA CAT ACG ACA ACT GC-3') and oTsr4 (5'-GTT CCA TGT GAA CAG CAG T-3') amplifies a region in the LSU-RNA known as the expansion segment V (ESV) (6). Larvae from reference strains of *T. spiralis* (code ISS3), *T. pseudospiralis* (code ISS13), and *T. britovi* (code ISS2) were used as controls.

Address for correspondence: Philippe Brouqui, Faculté de Médecine, 27 Boulevard Jean Moulin, F-13385 Marseille cedex 5, France; fax: 33-4-9183-0390; e-mail: Philippe.Brouqui@medecine.univ-mrs.fr.

Crude and excretory-secretory *Trichinella* antigens were prepared from larvae for enzyme-linked immunosorbent assay (ELISA) and immunoblotting (7). An indirect ELISA was used to detect parasite-specific immunoglobulin (Ig) G in human serum samples (8). Briefly, antigens were used at a concentration of 5 µg/ml in 0.1 M carbonate-bicarbonate buffer, pH 9.6. Serum samples were studied at several dilutions (range 1:200 to 1:6,400), and the conjugate (Bio-Rad, Hercules, CA) was at 1:10,000 dilution. Electrophoresis of crude and excretory-secretory antigens from *T. spiralis*, *T. pseudospiralis*, and *T. britovi* was done with 10% sodium dodecyl sulfate (SDS)–polyacrylamide gels under reducing conditions. Proteins were transferred to nitrocellulose and incubated with human sera diluted 1:100. Serum samples from five persons with confirmed trichinellosis and five known to be *Trichinella* free were used as positive and negative controls.

The incubation period for the hunters (patients 2 and 4), who both ate >300 g of boar meat, was half as long, and their clinical symptoms (fever and myalgias) lasted twice as long as those of the other two patients, who ate <300 g. Other clinical and laboratory abnormalities were not correlated with the estimated size of the inoculum. Diarrhea was the initial symptom for all patients (Table). When the patients were hospitalized on October 31, they had fever, asthenia, and myalgias (torn muscle pain worsened by exertion); none had vomiting or rash. The patients were treated with albendazole (800 mg/day) for 10 days combined with prednisone (30 mg/kg/day) for the first 3 days.

Table. Epidemiologic, clinical, laboratory, and outcome characteristics of four patients infected with *Trichinella pseudospiralis*, southeastern France

| | Patients | | | |
|---|---|---|---|---|
| Characteristics | 1 | 2 | 3 | 4 |
| Age (years) | 28 | 62 | 60 | 47 |
| Sex | M | M | F | M |
| Amount of boar meat eaten (g) | <300 | >300 | <300 | >300 |
| Incubation time (days) | 17 | 7 | 14 | 7 |
| Duration of fever (days) | 13 | 29 | 14 | 20 |
| Duration of myalgias (days) | 17 | 30 | 17 | 34 |
| Measured peak temperature (°C) | 39.8 | 39.8 | 38.6 | 39.2 |
| Arthralgias | No | No | No | Yes |
| Diarrhea | Yes | Yes | Yes | Yes |
| Vomiting | No | No | No | No |
| Periorbital edema | Yes | Yes | Yes | No |
| Conjunctivitis | No | Yes | No | No |
| Rash | No | No | No | No |
| Insomnia | Yes | No | No | No |
| Asthenia | Yes | Yes | Yes | Yes |
| Dizziness | Yes | No | No | No |
| leukocytes (N[b] = 4.0-10x$10^9$/L)[a] | 10.0 | 11.8 | 8.3 | 16.7 |
| Eosinophilia [peak] (N = 0-4.0x$10^9$/L) | 1.9 [2.3] | 3.1 [3.7] | 1.6 [3.1] | 5.3 [5.3] |
| Hemoglobin (N = 14-17 g/dL [men], 12-15 g/dL [women]) | 14.2 | 14.0 | 10.3 | 14.0 |
| Fibrinogen (N = 2.5-4.5 g/L) | 5.0 | 4.3 | 3.8 | 4.1 |
| Erythrocyte sedimentation rate (N = <10 mm/h) | 25 | 19 | 3 | ND[c] |
| Creatine kinase [peak] (N = <200 UI/L) | 5,874 [8,398] | 787 [2,670] | 4,277 [4,277] | 286 [286] |
| Lactate dehydrogenase (N = 100–620 UI/L) | 1,968 | 1,079 | 1,921 | 716 |
| Aspartate transaminase (N = <50 UI/L) | 22 | 61 | 186 | 27 |
| Alanine transaminase (N = <60 UI/L) | 90 | 61 | 212 | 47 |
| Triglycerides (N = 0.4-1.8 g/L) | 1.96 | 2.44 | 3.08 | 1.70 |
| Plasma total protein (N = 62–80 g/L) | 64 | 56 | 52 | 50 |
| Plasma albumin (N = 39–50 g/L) | 35 | 30 | 27 | 27 |

[a]Unless otherwise noted, laboratory findings were recorded at the time of admission to the hospital.
[b]N: Normal range.
[c]ND: Not determined.

The asthenia and myalgias initially worsened but then gradually improved, and all four patients recovered completely within 4 months.

All patients had elevated peripheral blood eosinophil counts (1.6 to 5.3 x $10^9$/L) and decreased plasma albumin levels. Serum creatine phosphokinase concentrations were elevated, with peak levels of 286 to 8,389 U/L (Table).

A frozen meat sample from the boar was highly infected (187 larvae/g), but all larvae were dead. Examination of muscle samples by the compression method showed that all larvae were nonencapsulated. Histologic examination of deltoid muscle tissue from biopsies performed on all four patients on November 3 showed active myositis with numerous necrotic fibers, inflammatory infiltrates of mononuclear cells, and *Trichinella* larvae (Figure 1). All sera analyzed by ELISA were positive at dilutions up to 1:6,400 with both crude and excretory-secretory antigens. The protein electrophoresis patterns observed with these larval antigens differed; however, serum samples from the four patients and control samples from persons infected with other *Trichinella* species (*T. spiralis* or *T. britovi*) recognized both antigens (prominent bands in the approximate range of 40 to 75 kD). The expansion segment V of larvae from the wild boar (310 bp) was identical by PCR to that of the *T. pseudospiralis* reference isolate (Figure 2).

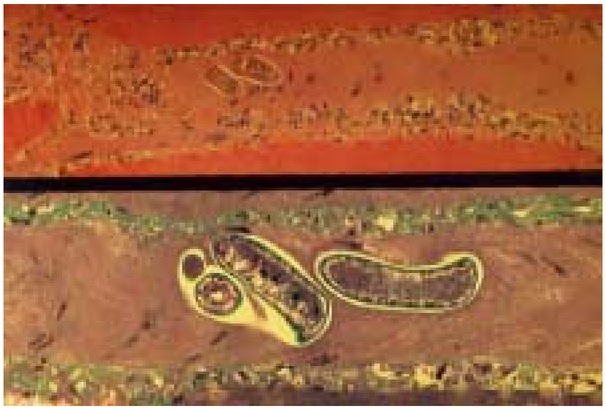

Figure 1. Sections of a muscle biopsy specimen from a patient infected with *Trichinella pseudospiralis* on day 32 after infection. The identified larva is nonencapsulated. Inflammatory cells are noted in the interstitium. Upper panel: hematoxylin and eosin stain at 50X magnification. Bottom panel: Masson trichrome stain at 100X magnification.

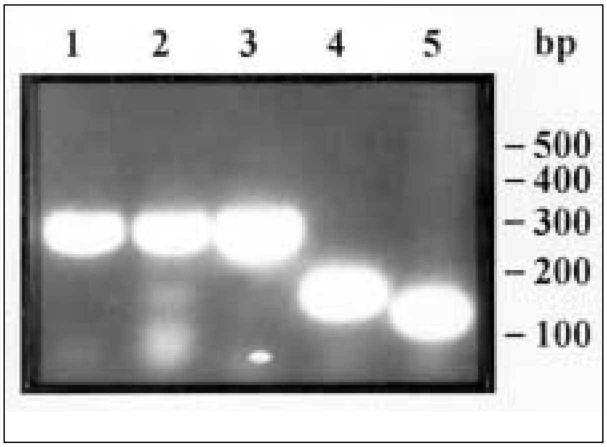

Figure 2. Gel agarose electrophoresis of the polymerase chain reaction amplification products of *Trichinella* sp. larvae. Lines 1 and 2, larvae in wild boar meat from Camargue, France; line 3, larva from the reference strain for *T. pseudospiralis*; line 4, larva from the reference strain for *T. spiralis*; and line 5, larva from the reference strain for *T. britovi*. Molecular weight markers: 50 base pairs (bp) DNA ladder (Pharmacia).

### Conclusions

This is the first report of human *T. pseudospiralis* infection in Europe. The first reported human case was detected in New Zealand, but the infection was probably acquired in Tasmania (9). This is only the third reported human *T. pseudospiralis* outbreak in the world. The first, in Thailand, affected 59 persons; one died (4). The second, in Kamchatka, Russia, affected approximately 30 persons (3). The clinical findings in our patients are consistent with previous reports of uncomplicated *T. pseudospiralis* (4) and *T. spiralis* infections (10). None of our patients had the life-threatening cardiopulmonary, renal, and central nervous system complications typical of trichinellosis infection. However, our patients had two unusual clinical features: fever persisted 13 to 29 days, considerably longer than previously reported for patients infected with *T. pseudospiralis* (4) or *T. spiralis* (10); and all four patients recovered completely within 4 months, in contrast with previous reports of severe asthenia and myalgias persisting for longer periods (4,11). Our patients received early treatment with effective anthelminthics and responded rapidly, which may explain the shorter duration of clinical symptoms. The laboratory findings in our four patients are consistent with those reported in trichinellosis; however, low plasma albumin and elevated triglyceride levels have not been reported in earlier outbreaks. The results of the Western blot analysis of sera from our patients demonstrated that immunoblotting cannot be used to identify the etiologic agent on the basis of recognized antigens. These findings contrast with those of a previous report (11) of an unusual Western blot pattern in serum samples from a patient thought to be infected only with *T. pseudospiralis*. However, subsequent investigation showed that this patient was also infected with another tissue nematode (*Haycocknema perplexum*)(12,13).

In Europe, *T. pseudospiralis* has been detected in a raccoon dog in the Krasnodar region of Caucasus (14), in two night birds of prey in central Italy (15), and recently in four raccoon dogs, one wild boar, and one brown rat in Finland (1). Although *T. pseudospiralis* can be considered a sylvatic genotype, the recent finding of this parasite in domestic pigs and brown rats on a farm in Kamchatka (3) suggests that, in certain epidemiologic situations, this parasite is transmitted to the human environment and should be considered a new potential parasite for domestic pigs. Pigs raised on ecologic (organic) farms are more likely to feed on infected wild animal carcasses than those raised on industrialized farms (16). These epidemiologic data suggest that either the prevalence of *T. pseudospiralis* infection is increasing in sylvatic and domestic animals of Europe and other continents or that techniques for diagnosing human and animal infections have improved, allowing identification of this nonencapsulated *Trichinella* species. Trichinelloscopy (visualizing *Trichinella* larvae by transillumination of small pieces of muscle from the diaphragm pillars between two thick slides) is used for trichinellosis screening in slaughterhouses. Because the collagen capsule is lacking, *T. pseudospiralis* larvae can easily be mistaken for muscle fiber. Therefore, trichinelloscopy is ineffective for screening in slaughterhouses or for diagnosis of human biopsy specimens. The finding that all larvae detected after artificial digestion of frozen meat were dead suggests that freezing may make game meat safe from *T. pseudospiralis* infection.

Experimental infection of domestic pigs and wild boars with *T. pseudospiralis* (17,18) showed that these hosts can harbor a substantial number of *T. pseudospiralis* larvae up to 20 weeks, but most muscle larvae had disappeared by 40 weeks after infection. However, several factors can contribute to the infectivity and persistence of this parasite species in domestic and sylvatic swine: the genetic variability of *T. pseudospiralis* isolates (6); the genetic variability of pigs and wild boars; and the occurrence in nature of stress, starvation, or concomitant infections that can induce immunosuppression in wild boars.

Twelve cases of trichinellosis associated with eating wild boar meat were reported from June 1994 to December 1995, in southeastern France, clustered around seven geographic foci (19). Some of these cases may have been caused by *T. pseudospiralis*, since neither histology of muscle biopsy nor recently developed molecular typing methods were used to verify the specific diagnosis. Molecular typing has opened new avenues for scientific investigations of trichinellosis and promises better understanding of the emerging pathogen that causes it. The broad spectrum of *T. pseudospiralis* hosts (both mammals and birds), the difficulty in detecting this parasite by trichinelloscopy, the potentially severe clinical picture in humans, and the increasing occurrence of this parasite indicate that expanded surveillance is needed to monitor the introduction and spread of trichinellosis.

### Acknowledgments
The authors thank J. Bakken for English review of the manuscript, M. Niang and S. Badiaga for their assistance, and J. Delmont for helpful comments.

Dr. Ranque is a specialist in infectious and tropical diseases at University Hospital in Marseille, France. His research focuses on the human host's genetic susceptibility to infectious diseases.

### References

1. Pozio E, Shaikenov B, La Rosa G, Obendorf DL. Allozymic and biological characters of *Trichinella pseudospiralis* isolates from free-ranging animals. J Parasitol 1992;78:1087-90.
2. Lindsay DS, Zarlenga DS, Gamble HR, Al-Yaman F, Smith PC, Blagburn BL. Isolation and characterization of *Trichinella pseudospiralis* Garkavi, 1972 from a black vulture (*Coragypus atratus*). J Parasitol 1995;81:920-3.

3. Britov VA. Trichinosis in Kamchatka. Wiad Parazytolo 1997;43:287-8.
4. Jongwutiwes S, Chantachum N, Kraivichian P, Siriyasatien P, Putaporntip C, Tamburrini A, et al. First outbreak of human trichinellosis caused by *Trichinella pseudospiralis*. Clin Infect Dis 1998;26:111-5.
5. Pozio E. Isoenzymatic typing of 23 *Trichinella* isolates. Trop Med Parasitol 1987;38:111-6.
6. Zarlenga DS, Aschenbrenner RA, Lichtenfels JR. Variations in microsatellite sequences provide evidence for population differences and multiple ribosomal gene repeats within *Trichinella pseudospiralis*. J Parasitol 1996;82:534-8.
7. Murrell KD, Anderson WR, Schad GA, Hanbury RD, Kazacos KR, Gamble HR, et al. Field evaluation of the enzyme-linked immunosorbent assay for swine trichinosis: efficacy of the excretory-secretory antigen. Am J Vet Res 1986;47:1046-9.
8. Pozio E, Varese P, Morales MA, Croppo GP, Pelliccia D, Bruschi F. Comparison of human trichinellosis caused by *Trichinella spiralis* and by *Trichinella britovi*. Am J Trop Med Hyg 1993;48:568-75.
9. Andrews JR, Ainsworth R, Abernethy D. *Trichinella pseudospiralis* in man. Lancet 1993;342:298-9.
10. Leclerc C, Marchou B, Sailler L, Bonnet E, Moron M, Alcayde S, et al. Une nouvelle épidémie de trichinellose: 117 cas en Midi-Pyrénées. Epidémiologie, aspects cliniques et traitement. Pathol Biol (Paris) 1999;47:573-5.
11. Andrews JR, Bandi C, Pozio E, Gomez Morales MA, Ainsworth R, Abernethy D. Identification of *Trichinella pseudospiralis* from a human case using random amplified polymorphic DNA. Am J Trop Med Hyg 1995;53:185-8.
12. Andrews JRH, Ainsworth R, Pozio E. Nematodes in human muscle. Parasitol Today 1997;13:488-9.
13. Spratt DM, Beveridge I, Andrews JR, Dennett X. *Haycocknema perplexum* n.g., n.sp. (Nematoda: Robertdollfusidae): an intramyofibre parasite in man. Syst Parasitol 1999;43:123-31.
14. Garkavi BL. The species of *Trichinella* isolated from wild carnivores. Veterinariia 1972;10:90-1.
15. Pozio E, Goffredo M, Fico R, La Rosa G. *Trichinella pseudospiralis* in sedentary night-birds of prey from Central Italy. J Parasitol 1999;85:759-61.
16. Pozio E. Factors affecting the flow among domestic, synanthropic and sylvatic cycles of *Trichinella*. Vet Parasitol. In press 2000.
17. Kapel CMO, Gamble HR. Infectivity, persistence, and antibody response to domestic and sylvatic *Trichinella spp.* in experimentally infected pigs. Int J Parasitol 2000;30:215-21.
18. Kapel CMO. Experimental infections with sylvatic and domestic *Trichinella spp.* in wild boars: infectivity, muscle larvae distribution, and antibody response. J Parasitol. In press 2001.
19. Dupouy-Camet J, Allegretti S, Truong T. Enquête sur l'incidence de la Trichinellose en France (1994-1995). Bull Epidemiol Hebdom 1998;28:122-3.

## References

[PDF_00357] Andrews J. R., Ainsworth R., Abernethy D. (1993). Trichinella pseudospiralis in man.. Lancet.

[PDF_00368] Andrews J. R., Ainsworth R., Pozio E. (1997). Nematodes in human muscle.. Parasitol Today.

[PDF_00363] Andrews J. R., Bandi C., Pozio E., Gomez Morales M. A., Ainsworth R., Abernethy D. (1995). Identification of Trichinella pseudospiralis from a human case using random amplified polymorphic DNA.. Am J Trop Med Hyg.

[PDF_00374] Garkavi B. L. (1972). Vid trikhinelly, vydelennyi ot dikikh khishchnykh.. Veterinariia.

[PDF_00337] Jongwutiwes S., Chantachum N., Kraivichian P., Siriyasatien P., Putaporntip C., Tamburrini A., La Rosa G., Sreesunpasirikul C., Yingyourd P., Pozio E. (1998). First outbreak of human trichinellosis caused by Trichinella pseudospiralis.. Clin Infect Dis.

[PDF_00382] Kapel C. M., Gamble H. R. (2000). Infectivity, persistence, and antibody response to domestic and sylvatic Trichinella spp. in experimentally infected pigs.. Int J Parasitol.

[PDF_00359] Leclerc C., Marchou B., Sailler L., Bonnet E., Moron M., Alcayde S., Servat M., Duchen C., Hemery C., Magnaval J. F. (1999). Une nouvelle épidémie de trichinellose: 117 cas en Midi-Pyrénées. Epidémiologie, aspects cliniques et traitement.. Pathol Biol (Paris).

[PDF_00328] Lindsay D. S., Zarlenga D. S., Gamble H. R., al-Yaman F., Smith P. C., Blagburn B. L. (1995). Isolation and characterization of Trichinella pseudospiralis Garkavi, 1972 from a black vulture (Coragyps atratus).. J Parasitol.

[PDF_00348] Murrell K. D., Anderson W. R., Schad G. A., Hanbury R. D., Kazacos K. R., Gamble H. R., Brown J. (1986). Field evaluation of the enzyme-linked immunosorbent assay for swine trichinosis: efficacy of the excretory-secretory antigen.. Am J Vet Res.

[PDF_00376] Pozio E., Goffredo M., Fico R., La Rosa G. (1999). Trichinella pseudospiralis in sedentary night-birds of prey from Central Italy.. J Parasitol.

[PDF_00341] Pozio E. (1987). Isoenzymatic typing of 23 Trichinella isolates.. Trop Med Parasitol.

[PDF_00324] Pozio E., Shaikenov B., La Rosa G., Obendorf D. L. (1992). Allozymic and biological characters of Trichinella pseudospiralis isolates from free-ranging animals.. J Parasitol.

[PDF_00353] Pozio E., Varese P., Morales M. A., Croppo G. P., Pelliccia D., Bruschi F. (1993). Comparison of human trichinellosis caused by Trichinella spiralis and by Trichinella britovi.. Am J Trop Med Hyg.

[PDF_00370] Spratt D. M., Beveridge I., Andrews J. R., Dennett X. (1999). Haycocknema perplexum n. g., n. sp. (Nematoda: Robertdollfusidae): an intramyofibre parasite in man.. Syst Parasitol.

[PDF_00343] Zarlenga D. S., Aschenbrenner R. A., Lichtenfels J. R. (1996). Variations in microsatellite sequences provide evidence for population differences and multiple ribosomal gene repeats within Trichinella pseudospiralis.. J Parasitol.

